# Adsorption of chromium from electroplating wastewater using activated carbon developed from water hyacinth

**DOI:** 10.1186/s13065-023-00993-4

**Published:** 2023-07-24

**Authors:** Zemene Worku, Samuel Tibebu, Jemal Fito Nure, Solomon Tibebu, Welldone Moyo, Abera Demeke Ambaye, Thabo T. I. Nkambule

**Affiliations:** 1 Department of Environmental Engineering, Addis Ababa Science, and Technology University, 16417, Addis Ababa, Ethiopia; 2 Institute for Nanotechnology and Water Sustainability (iNanoWS), University of South Africa, Science Campus Florida, Johannesburg, South Africa

**Keywords:** Adsorbent, Industrial effluent, Experimental design, Heavy metal, Pollution, Water treatment

## Abstract

Industrial wastewater polluted with high concentrations of Cr is commonly discharged into water resources without proper treatment. This gives rise to the deterioration of water quality and imposes adverse effects on public health. Therefore, this study is aimed at removing Cr from electroplating wastewater using activated carbon produced from water hyacinth under a full factorial experimental design with three factors and three levels (pH,2,5 and 8, adsorbent dose 0.5,1and1.5 in 100 mL and contact time 30, 60 and120 min). A phosphoric acid solution of 37% was used to activate the carbon, which was then subjected to thermal decomposition for 15 min at 500 °C. The activated carbon was characterized by the presence of a high surface area (203.83 m^2^/g) of BET, cracking of adsorbent beads of SEM morphology, amorphous nature of XRD, and many functional groups of FTIR such as hydroxyl (3283 cm^−1^), alkane (2920 cm^−1^), nitrile (2114 cm^−1^) and aromatics (1613 cm^−1^). The minimum Cr adsorption performance of 15.6% was obtained whereas maximum removal of 90.4% was recorded at the experimental condition of pH 2, adsorbent dose of 1.5 g/100 mL, and contact time of 120 min at a fixed value of initial Cr concentration of 100 mg/L. Similarly, the maximum Cr removal from real electroplating wastewater was 81.2% at this optimum point. Langmuir's model best described the experimental value at R^2^ 0.96 which implies the adsorption is chemically bonded, homogeneous, and monolayer. Pseudo-second-order model best fits with the experimental data with R^2^ value of 0.99. The adsorbent was regenerated for seven cycles and the removal efficiency decreased from 93.25% to 21.35%. Finally, this technology is promising to be scaled up to an industrial level.

## Introduction

The discharge of untreated wastewater with heavy metals into the environment can harm both the ecosystem and public health. Water pollution is increasing rapidly from time to time and contributing significantly to water stress and scarcity whereas the global water demand is increasing rapidly which will be estimated to be 5440–6900 billion m^3^ by 2050. Water stress hit the life of over 2 billion people which will rise to 40% of the global water deficit by 2030. Contamination of freshwater bodies with chemicals is rapidly increasing but the knowledge of its impacts is still scant. Globally, more than 80% of all industrial and municipal wastewater is discharged into the environment without prior treatment [1]. The big challenge to water quality and sustainability will be the irresponsible discharge of pollutants into the environment [2]. The chemistry and toxicity levels of the heavy will be unknown in the long run and the potential to degrade public health and the environment would be severe [2]. However, exposure to micropollutants including heavy metals can result in mutations, endocrine disruption, a congenital disorder, dermal pathologies, high toxicity, carcinogenicity, reproductive impairments, neurobehavioral disorders, and physical abnormalities [3–6]. In line with this, electroplating wastewater is among many industrial wastewaters, which contains a high concentration of Cr that can cause a huge health threat to the public and the environment. Normally, the electroplating technique comprises several activities like acid pickling, alkaline cleaning, plating, and rinsing which results in a huge amount of heavy metals, cyanides, nitrates, and sulfate complexes in the wastewater [7]. Particularly, the concentration of Cr in electroplating wastewater is high. Similarly, Cr is the most consumable about 90% of the tanning process in the leather industries. However, Cr is a toxic and abundant chemical in many industries across the globe [8]. Cr is the primary chemical used in the chrome process method and many unit operations used in the industry [9, 10]. Severe environmental pollution of chrome is highly associated with the discharge of the Cr saturated industrial wastewater including the electroplating wastewater [11]. In addition to Cr, other harmful chemicals such as phthalates, grease, organic chemicals (tannins), sulfonated oils, azo dyes, surface-active compounds, and phenolic compounds have been commonly used in many industrial processes [12]. This is significantly contributing to the toxicity of industrial wastewater. Cr exists in different forms such as chromate (CrO_4_^2–^, Cr(OH)^2+^, Cr(OH)_3_^◦^, and Cr(OH)_4_^−^) and dichromate (Cr_2_O_7_^2–^, HCrO_4_^−^, and CrO_4_^2−^ which showed the complex chemistry of the Cr. This, in turn, creates a complex aquatic environment with severe long-run impacts on public and environmental problems [13, 14]. Moreover, many public health problems such as mutagenic, anemia, kidney dysfunction, carcinogenic, diarrhea, skin irritation, liver failure, lung cancer, and vomiting [12, 13, 15, 16]. Currently, the toxicity and hazardous nature of the Cr have escalated to a high level of toxicity including genotoxicity, cytotoxicity, and phytotoxicity. However, the level of toxicity of the Cr is varied based on the oxidation number. For instance, Cr (VI) is 500 times more toxic than Cr (III) [17]. Generally, Cr disposal management is essential before the discharge of the effluent from tannery industrial wastewater.

Conventional wastewater treatment processes are not sufficiently eliminated heavy metals including Cr [18]. But, advanced water treatment technologies such as membrane separation processes, advanced oxidation processes, ozonation, adsorption, and membrane bioreactors achieved high Cr removal [6, 19–21]. Recently, advanced wastewater treatment technologies are considered the state of the art in heavy metal removal [22]. These technologies have many limitations including highly skilled men, expensive, massive sludge production, possessing pollutants by-products, and intensive consumption of the chemical, and energy. These situations make the technologies inconvenient for developing countries. However, adsorption is considered effective, efficient, convenient for upgrading, simple to design, and eco-environmentally practically [23]. Particularly, the performance of activated carbon is excellent but its wide application is limited due to the high production cost [24]. Hence, searching for low-cost, efficient, and locally available materials is still an ongoing process. Normally, the selection of precursor materials and the production of perfect adsorbents is a challenging topic in the adsorption industry. Materials sustainability and regeneration of adsorbents are a big challenge in the water treatment sector. These adsorbents are supposed to give the way to rehabilitate, reuse and recycle to ensure sustainability [25]. Essentially, an ideal adsorbent should be available easily, non-soluble, efficient, easily manufactured, non-toxic, cost-effective, eco-friendly, and regenerate effortlessly [26]. Among many adsorbent groups, laboratory-based activated carbon is promising and many efforts have been made for the water and wastewater purification department. Hence, searching for different kinds of adsorbents and activating methods are essential components of the adsorption processes. In line with this, many activated carbons produced from locally available materials such as bamboo, coconut husks, willow peat, wood, lignite, coal, water hyacinth, and petroleum pitch have been reported [23, 27, 28]. Some of these activated carbons have been applied for Cr from aqueous solutions and wastewater [21, 29–32]. Generally, for water purification use water hyacinth as an adsorbent material.

Water hyacinth (*Eichhornia crassipes)* floating macrophyte with a high density of 60 kg/m^2^ and height of 1 m. Water hyacinth is a plant that is grown in many climatic conditions including in Asia, Australia, Africa, Europe, North America, and southeast Asian countries. This plant can be considered an invasive and nuisance plant that can shade aquatic ecosystems, block water flow and reduce the concentration of dissolved oxygen in the water. Normally, water hyacinth is the most problematic aquatic weed in the world with uncontrolled spreading across the water bodies outside its native area [33]. Globally, water hyacinth is a huge threat to water bodies and the survival of aquatic organisms. Recently, water hyacinth is using as a bio-sorbent that is composited of carbon and oxygen mostly chemically [34]. This biosorbent was modified chemically through alkali (NaOH), acid, and peroxide and has been proven to enhance the efficacy of the adsorbents. Water hyacinth biosorbent can also be modified by using the quaternary ammonium salt, N-Cetyl-N, N, N–trimethyl ammonium bromide for the decolorization of the hazardous textile dye [35]. The biochar of this plant was effective in the removal of heavy metals like Cd which was reported in the range of from 24.2 to 45.8 mg/g even though it can reach up to 70 mg/g [36]. The maximum adsorption capacities of biochar of water hyacinth for Ni^2+^ 28.6 ± 3.9, Zn^2+^ 18.9 ± 1.6, and Pb^2+^ 76.8 ± 4.7 mg/g at 25.0 °C [33]. A pair of electrons on the oxygen of activated carbon can be shared with the cation of the heavy metals which enhances the adsorption process reflected by the removal of 95% lead (II) at initial concentrations of 100 mg/L [37]. Similarly, the adsorption of phosphorus through this biochar was fostered efficiently up to 60 mg P/g indicating that water hyacinth is a potential adsorbent to mitigate water pollution [38]. The application of water hyacinth biochar for wastewater treatment reduced the concentrations of Cl^−^, BOD_5_, COD, and Cr III by 56, 93.4, 92.6, and 99%, respectively [39]. The applications of water hyacinths for the treatment of water bodies are the balance between water purification and enhancing the health of the aquatic species. This is a good opportunity that can convert the nuisance-invading species into adsorbent material. But, the study of water hyacinths activated carbon under various surface functionalities and preparation methods for Cr removal and treatment optimization was limited in the literature. Therefore, this study aimed to remove chromium from electroplating wastewater using activated carbon from water hyacinth under a full factorial experimental design using adsorbent dose, pH, and contact time. The optimization and interaction of adsorption were performed through three factors at the three-level full factorial approaches which can reduce the number of experiments, time utilized, better treatment performance, and resource consumption.

## Materials and methods

### Wastewater sampling and characterization

**Fig. 1:**
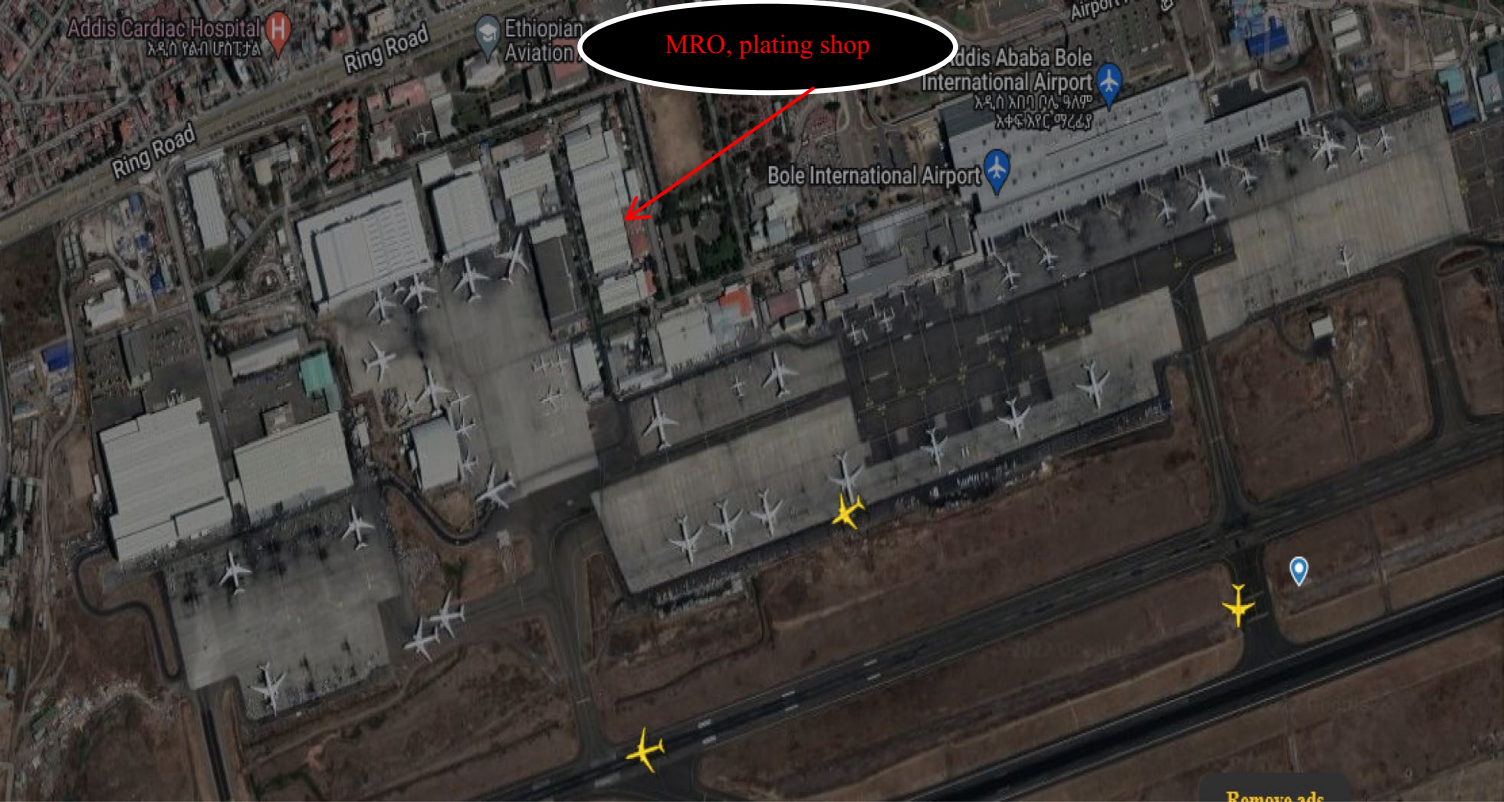
Ethiopian airlines MRO, Plating Shop location at the Bole international airport

Wastewater sample was collected from the plating and cleaning shop of Ethiopian airlines MRO which is located at Addis Ababa Bole International Airport serves. This area is situated at a geographical coordinate of 8^o^58′40’’N, and 38^o^47′57″ E which is indicated in Fig. 1. Ethiopian Airlines is an Ethiopian flag carrier wholly owned by the Ethiopian government. Ethiopian Airlines was founded on December 1945 and commenced on 8 April 1946 which was expanded to an international flight in 1951. Represent composite wastewater samples were collected three times a day and thoroughly mixed. The sample was collected in polyethylene plastic bottles which were washed with detergent and diluted acid and finally rinsed with distilled deionized water. Some of the water samples were analyzed immediately on site for a few physicochemical parameters such as temperature, electric conductivity, and pH whereas the rest parameters were analyzed at the laboratory after the samples were transported to Addis Ababa Science and technology university and stored in the refrigerator at 4 °C until the analyses were performed. Normally, the sampling of the physicochemical parameter was duplicated and triplicate analysis was carried out. Particularly, the physicochemical parameters such as temperature, electric conductivity, COD, TS, and Cr used standard methods for the examination of water and wastewater and the results of these analyses were reported in the form of the mean plus standard deviations [23, 28, 40, 41].

### Adsorbent preparation and characterization

#### Preparation of activated carbon

**Fig. 2:**
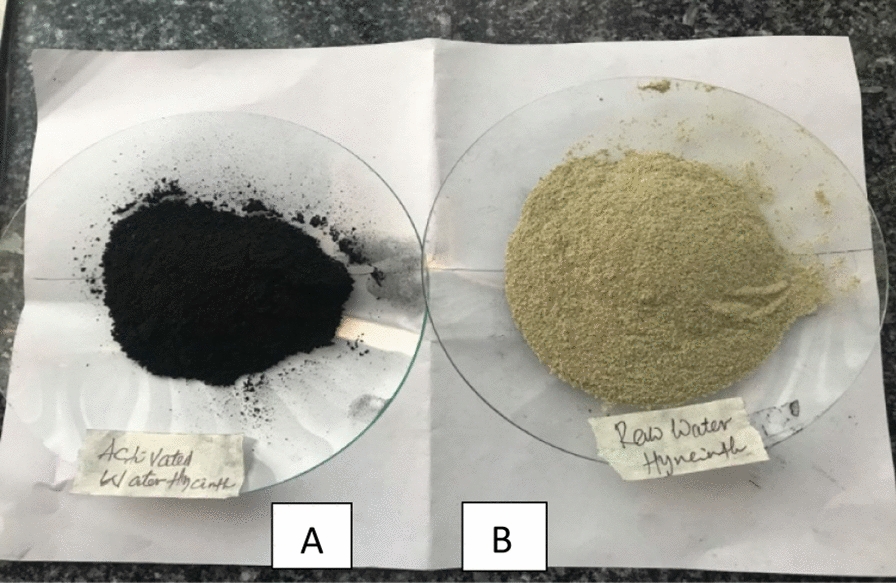
Water hyacinth activated carbon **A** and raw water hyacinth adsorbent **B**

The water hyacinth samples were collected from Lake Denbel and then the plant sample was washed with distilled water several times and dried at low room temperature. The water hyacinth sample was dried properly in the oven at a standard temperature of 70 °C for 24 h. The dried sample was chopped up at different meshes sizes of 125 to 150 μm and the size distribution was arranged using sieve separation. The prepared activated and raw adsorbent was indicated in Fig. 2. The dried water hyacinth sample was activated using 37% phosphoric acid solution in the ratio of 1:1 power of water hyacinth (mg) to phosphoric acid (mL) [28]. The sample was properly soaked in a solution for 24 h at room temperature. Then, the sample was washed thoroughly and dried in an oven at 110 ± 5 °C. This adsorbent material was thermally activated at a temperature of 500 °C for 15 min in a muffle furnace [21]. Finally, after cooling the adsorbent material, it was deposited in a plastic bag and stored in the desiccator until it was used for the adsorption process [21, 28, 30, 31].

#### Adsorbent characterization

##### Proximate analysis

Proximate analysis such as the ash content, moisture content, volatile matter, and fixed carbon of activated carbon was performed according to the American Society for Testing and Materials. The moisture content of activated carbon was carried out by heating a 1.0 g adsorbent sample in an oven at 110 °C for 2 h and the moisture lost was calculated in percentage. Similarly, the volatile matter of activated carbon was determined after heating 1 g of adsorbent in the muffle furnace at 800 °C for 8 min. Thirdly, the ash content of the adsorbent was calculated as the amount of the residue after thorough heating of 1 g of adsorbent at 500 °C for 4 h. Finally, the fixed carbon of the activated carbon was calculated by subtracting the sum of all the percentages of the amount of moisture, ash content, volatile matter, and fixed carbon from 100%. The percentage of moisture content, volatile matter, Ash content, and fixed carbon were indicated in Eqs. (1), (2), (3), and (4), respectively.

1 $$MC(\mathrm{\%})=\frac{{W}_{1}- {W}_{2} }{{W}_{1}}*100\mathrm{\%}$$

2 $$\mathrm{VM }=\frac{{W}_{1}- {W}_{2} }{{W}_{1}}*100\%$$

3 $$\mathrm{AC }(\mathrm{\%}) = \frac{{W}_{2}}{{W}_{1}}*100$$

4 $$FC (\mathrm{\%}) = 100\mathrm{\% }- (\mathrm{MC\% }+\mathrm{ VM\% }+\mathrm{ AC \%})$$

where MC is the moisture composition of activated carbon in percentages, *W*_1_ is the weight of activated carbon before the application of thermal (g) and *W*_2_ is the weight of the adsorbent sample after thermal drying (g), VM, of volatile matter, AC represents Ash content and FC is the fixed carbon in adsorbent materials [42].

##### The pH of the point of zero charges (${{\varvec{p}}{\varvec{H}}}_{{\varvec{p}}{\varvec{z}}{\varvec{c}}}$)

The surface charge of the adsorbent materials is measured using the pH of the point of zero charges (${pH}_{pzc}$). The measurement of ${pH}_{pzc}$ was performed using 1 g of activated water hyacinth adsorbent which was placed in 50 mL solution in Erlenmeyer flasks for the time of 48 h and the pH of the Solution was adjusted using the solution of 0.01 M HCl and 0.01 M NaOH in the range of the 2 to 12 pH. Finally, the value of the ${pH}_{pzc}$ was determined from the intercept of pH final and pH initial curves [35, 43].

##### Scanning electron microscope (SEM)

One of the essential characterizations of the adsorbent materials is surface morphology. The surface morphology of the adsorbent was studied using the SEM analysis which is named JCM-6000PLUS BENCHTOP SEM, JOEL, Japan. The specific analysis was carried out under conditions that depend on the intention of the analysts. These specifications are the magnification, working distance, voltage irradiated, probe utilization, and secondary electron emission. The readymade adsorbent powder sample was placed on coated carbon tape of the sample holder. Then the adsorbent sample was characterized at operated at a current of 10 A working distance of 8 mm and operating energy of 15 kV with 1000 ×magnification [23, 28, 44].

##### Fourier transform infrared (FTIR) spectroscopy

The surface functional groups of the adsorbent are very essential to understanding the surface interaction between the adsorbent and adsorbate material in water solution. The functional groups of the adsorbent before and after adsorption were evaluated using Fourier Transform Infrared spectroscopy (FTIR, Thermo Nicolet 5700, and Waltham, MA, USA). The black adsorbent material is not conducive to analysis of the function groups and then adjusted to transparent using KBr. Hence, the adsorbent to KBr ratios was fixed at 2:200 which was thoroughly mixed to form a homogeneous mixture. This mixture was further grounded and an adsorbent pellet was formulated. This pellet was analyzed using FTIR spectroscopy at the range of wavelength of 500–4000 cm^−1^. Accordingly, the most common scanning rate in many researchers was 32 times/min and resolution can be varied according to the interest of the researcher [23, 35].

##### Brunauer–Emmett–Teller (BET)

The most common method for the specific surface area of the adsorbent is Brunauer–Emmett–Teller (BET). This process is performed through the adsorption and desorption of liquid nitrogen at 700 mm atmospheric pressure. This experiment was carried out using the BET surface area analyzer (Horiba instrument Inc. SA-9600). Normally, the adsorption–desorption was operated at − 196.5 °C of liquid nitrogen. But, the vacuum condition of adsorbent degassing temperature was performed at 150 $^\circ{\rm C}$. Finally, the value of the BET-specific surface area of the adsorbent before and after adsorption was calculated using the p/p0 ratio of the isothermal graph [23, 45].

##### X-ray diffraction (XRD)

The XRD technique is used to identify the crystalline or amorphous nature of the adsorbent materials. The specific machine used in this experiment was the X-ray powder diffraction (XRD) instrument (XRD-X-ray tube Cu 40 kv, 40 mA, Olympus BTXH) which was described by tube cu40 kV, 40 mA. The adsorbent powder analysis was operated at a Cu Kα source (λ = 1.54178 Å) and a scanning rate of 1 $^\circ$/min in the range of 10–70 $^\circ of$ 2θ through the continuous measuring mode. Moreover, the experiment was carried out using voltage and current of 15 kV and 5 mA at a fixed wavelength of 1.541 nm throughout the analysis. The peak observed during the analysis was used to differentiate the nature of the adsorbent [23, 27, 46].

### Chromium adsorption optimization

**Fig. 3:**
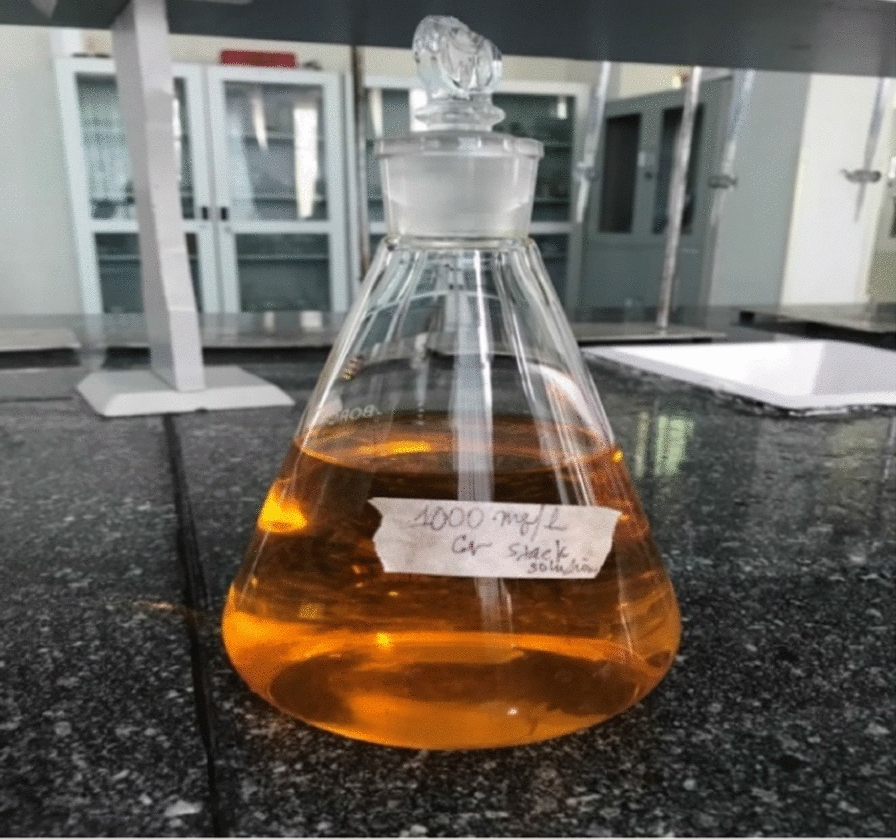
The stock solution of chromium (IV)

Chromium (IV) solution was prepared by dissolving 2.82 g of K_2_Cr_2_O_7_ in 1 L of distilled water 1000 mg/L Cr stock solution as indicated in Fig. 3. The working solution of chromium was prepared through a dilution process. The value of the Cr concentration was fixed according to the experimental design indicated in Table 1 [31, 32]. This experimental value was fixed based on the experimental and literature documents. a full factorial experimental design with three factors and three levels described as pH,2,5 and 8, adsorbent dose 0.5,1 and 1.5 in 100 mL, and contact time 30, 60, and 120 min.

**Table 1:** Factors and corresponding levels for full factorial experiment design

S.No	Variables	Low (− 1)	Middle (0)	High (+ 1)
1	Adsorbent dosage (g/100 mL)	0.5	1	1.5
2	pH	2	5	8
3	Contact time (min)	30	60	120

The Cr adsorption process was performed at room temperature under batch mode delivery. The removal efficiency of adsorbent material was calculated using removal percentage. Many adsorption experiments were performed differently [21, 31, 32]. The adsorption process was a full-factorial experimental design 3^3^ which resulted in 27 runs. The experimental numbers of the study were reduced to 20 runs based on the experiment number reduction mechanism (Box-Behnken design). The upper, middle, and lower values of the factors were designated with “− 1”, ‘‘0’’ and ‘‘ + 1’’, respectively. In this analysis, the Cr removal was the dependent variable whereas the factors such as adsorbent dose, pH, and contact time were independent variables. As a rule, the order of the experiment was arranged and performed based on a random selection method. Response surface methodology (RSM) was used to study the adsorption interactions. Normally, RSM is a collection of mathematical and statistical methods that are useful for the modeling and analysis of engineering problems. The effect of the main and interaction was investigated intensively.

The efficiency of the adsorbent was calculated using Eqs. 5, 6. The adsorption efficiency and capacity of the adsorbent (q_e_) were the most commonly used equation in adsorption evaluation tasks [23, 28, 42].

5 $$\mathrm{R}\left(\mathrm{\%}\right)=\left(\frac{{\mathrm{C}}_{\mathrm{i}}-{\mathrm{C}}_{\mathrm{f}}}{{\mathrm{C}}_{\mathrm{i}}}\right)*100$$

6 $${\mathrm{q}}_{\mathrm{e}}=\left(\frac{{\mathrm{C}}_{\mathrm{o }}-{\mathrm{C}}_{\mathrm{e}}}{\mathrm{m}}\right)*\mathrm{V}$$

where R is the Cr removal percentage, C_i_ is the initial Cr concentration (mg/L), C_o_ (mg/L) is the initial Cr concentration, V (L) is the volume of the solution, m (mg) is the mass of the adsorbent utilized, C_e_ (mg/L) is the equilibrium Cr concentration, and C_f_ is the final Cr concentration (mg/L) after the treatment.

### Adsorption isotherms

Adsorption isotherms were evaluated using initial chromium concentrations of 60, 80, 100, 120, and 140 mg/L at the fixed values of the adsorbent dose of 1.5 g/100 mL, contact time 120 min, solution, and pH 2. The two most common adsorption isotherms were used in this adsorption study. These two isotherms are Langmuir and Freundlich's models were used in many analyses of water and wastewater. These two models are the most commonly used adsorption isotherms in water and wastewater. The assumption behind the Langmuir isotherm is that the adsorbate accumulation is a monolayer for all binding sites whereas the Freundlich isotherm designates for multiplayer adsorption. Finally, the simplified and linearized forms of the Langmuir and Freundlich isotherms were shown in Eqs. (7), (8), (9), respectively.

7 $$\frac{\mathbf{C}\mathbf{e}}{\mathbf{q}\mathbf{e}}=\frac{\mathbf{C}\mathbf{e}}{{\mathbf{q}}_{\mathbf{m}\mathbf{a}\mathbf{x}}}+\frac{1}{{\mathbf{K}}_{\mathbf{L}}{\mathbf{q}}_{\mathbf{m}\mathbf{a}\mathbf{x}}}$$

8 $$\mathbf{R}\mathbf{L}=\frac{1}{1+{\mathbf{K}}_{\mathbf{L}}{\mathbf{C}}_{\mathbf{e}}}$$

9 $${\text{log}}\,{\text{q}}_{{\text{e}}} {\text{ = log}}\,{\text{KF + }}\frac{{1}}{{\text{n}}}{\text{log}}\,{\text{C}}_{{\text{e}}}$$

where q_max_ (mg/g) is the maximum adsorption capacity, Co (mg/L) is the initial concentration, K_L_ (L/mg) is the Langmuir constant, K_F_ indicates adsorption capacity (mg/g), 1/n is an empirical parameter related to the intensity of adsorption indicating favorable conditions if its value is between 0.1 and 1, Ce (mg/L) is the equilibrium values of initial concentration, q_e_ (mg/g) is the adsorption capacity at equilibrium, the R_L_ is the dimensionless, separation constant of Langmuir adsorption, favorable adsorption, the R_L_ value should be in between 0 and 1; for unfavorable adsorption, the R_L_ value is greater than 1, whereas RL values equal to 1 or 0 indicate linear and irreversible adsorption processes, respectively [45].

### Adsorption kinetics

Pseudo-first-order and pseudo-second-order models were used to determine the adsorption kinetics parameters at a contact time of 30, 45, 60, 90, and 120 min. The study was conducted at a fixed value of pH 2, an adsorbent dose of 1.5 g/100 mL, and an initial chromium concentration of 60 mg/L. The linearized forms of Pseudo-first-order and pseudo-second-order kinetics models are shown in Eqs. (10), (11) respectively.

10 $$\mathrm{log}(qe-{q}_{t})=\mathrm{log}qe-\frac{K1t}{2.303}$$

11 $$\frac{t}{ qt }=\ (\frac{t}{qe})+ \frac{1}{{{K}_{2}}_{{q}_{e}^{2}}}$$

where *qt* is the amount of Cr (III) on the surface of the activated carbon at time t (mg g^−1^) K2 is the rate constant of the pseudo-second-order adsorption (g/mg min) and *k*1 is the equilibrium rate constant of the pseudo-first-order adsorption (min^−1^).

### Regeneration study

The adsorbent regeneration and reusability were investigated by using 1 M of NaOH as a desorbing solution. A 3 g of saturated adsorbent is added to 150 mL NaOH solution and agitated by a mechanical stirrer for 120 min at 400 rpm at room temperature (25 $^\circ{\rm C}$). The filter paper was used to separate the regenerated adsorbent from the desorbing solution. Then, the adsorbent was washed with distilled water up to pH 7. The adsorbent was recycled for cations adsorption for seven successive cycles.

## Results and discussion

### Physicochemical properties of electroplating wastewater

The physicochemical properties of electroplating wastewater were investigated thoroughly and the values of the findings are present in Table 2. The values of each parameter are presented in the form of means plus standard deviation to check the variation among replications and duplicate measurements. In line with this, the average temperature and wastewater pH was found to be 21.00 ± 0.75 ℃ and 3.12 ± 0.81, respectively. These two wastewater parameters are decisive to influence the biochemical reaction of water and soil in addition to wastewater treatment performances. According to Ethiopian industrial effluent discharging standards, the maximum permissible discharging limits of wastewater in terms of temperature should be lower than 40 ℃ and the pH has to be within the range of 6–9. In terms of temperature, the wastewater is safe and can be discharged into the environment without further treatment intervention. However, this very acidic wastewater can’t be released into the environment without treatment. The discharge of such wastewater can affect the biochemical reaction of aquatic organisms, enhance the acceleration of the reaction rate for various chemical reactions in water bodies, increasing the solubility and complexity of organic and inorganic chemicals. The property of the wastewater showed that either dilution or chemical neutralization process is very essential before discharge or treatment. Total solid and EC of wastewater were found to be 351.64 ± 9.60 mg/L and 27.25 ± 0.23 mS/cm. Total solid is attributed to the dissolved and filtrate which can block the transparency of water bodies and reduce the amount of dissolved oxygen that endangers the survival of aquatic life through perturbation of normal interaction of living organisms and biochemical reactions whereas electrical conductivity is associated with the presence of total ions with charge numbers( mono-, di-, tri- and tetra charged ions) [20, 23]. Generally, such high concentrations of total solid matter can also impact the physicochemical and biological properties of soil and water bodies.

**Table 2:** The average values of physicochemical characteristics of electroplating wastewater

Parameters	Values
pH	3.10 ± 0.81
EC (mS/cm)	27.25 ± 0.23
Temperature ( C)	21.05 ± 0.156
BOD_5_ (mg/L)	316.25 ± 3.30
COD (mg/L)	750.87 ± 8.21
TS (mg/L)	351.00 ± 9.60
Cr (mg/L)	92.16 ± 0.768

The presence of organic matter is indicated by the high presence of a high concentration of COD and BOD_5_. But, the impact on wastewater treatment and the environment is described by the ratio of the BOD_5_/COD which is called the biodegradability index (BI). This biodegradability is can provide a lot of information about the selection of the right treatment and treatment plant. BI of 0.6, between 0.3 and 0.6, and below < 0.3 to indicate effectiveness, acclimatization required, and difficulty to go for biological treatment, respectively. Hence, the calculated value of BI in electroplating wastewater was about 0.43 which indicated that acclimatized microbial is mandatory in case the biological treatment plants use it. The average concentration of chromium recorded throughout the study period was 92.38 ± 0.768 mg/L. The high concentration of chromium in the wastewater is attributed to the amount of chromium consumed in the electroplating process particularly in new landing gear components and worn-out parts to prevent corrosion. The World Health Organization set the maximum permissible Cr (VI) concentration in potable water at 0.05 mg/L. The maximum permissible Cr (VI) concentration in potable water and surface water is 0.1 mg/L whereas industrial effluent discharging limits 0.25 mg/L according to EPAs of many nations [28]. Chromium concentration in electroplating wastewater is exceeding the maximum permissible discharging limits. The high risk of exposure to the high concentration of chromium is associated with its high solubility and migrant ion in the water solution Exposing this high concentration of chromium to the nearby environment and public health can cause kidney dysfunction, pulmonary congestion, skin irritation, hepatitis, anemia, diarrhea, and vomiting [47].

### Characterization of adsorbent

#### Proximate analysis

The organic carbon and inorganic components of the adsorbent were evaluated using proximate analysis. The weight percentages of the adsorbent were explained in terms of moisture, ash, fixed carbon, and volatile contents and presented in Table 3. Normally, a good adsorbent to be with a high percentage of fixed carbon and low composition of ash content. Moreover, the low moisture and volatile compositions of activated carbon are other good properties of a quality adsorbent. The fixed carbon is calculated based on the composition differences between 100% and the rest composition. The fixed carbon of this study was found to be 63.73% which indicated a good composition in line with many locally prepared adsorbents of the activated carbon. Thus, the activated carbon prepared from water hyacinth has a great potential to be scaled up for fabrication of the commercial activated in line with these specific parameters. In general, the composition of the proximate analysis was found to be in line with the finding of the study showing moisture content of 4.620%, ash at 36.0%, fixed carbon at 42%, and volatile matter at 10.43% [48]. To be a good adsorbent of activated carbon, the least component of the fixed carbon is 60% and above [49]. Similar values of the fixed carbon of locally prepared activated carbon were reported [28, 42, 50].

**Table 3:** The proximate composition of activated carbon of water hyacinth in terms of the weight percentages

Proximate analysis	Mass%
Moisture	6.30
Volatile matter	13.40
Ash content	19.17
Fixed carbon	63.73

#### The pH point zero charge (pHpzc)

**Fig. 4:**
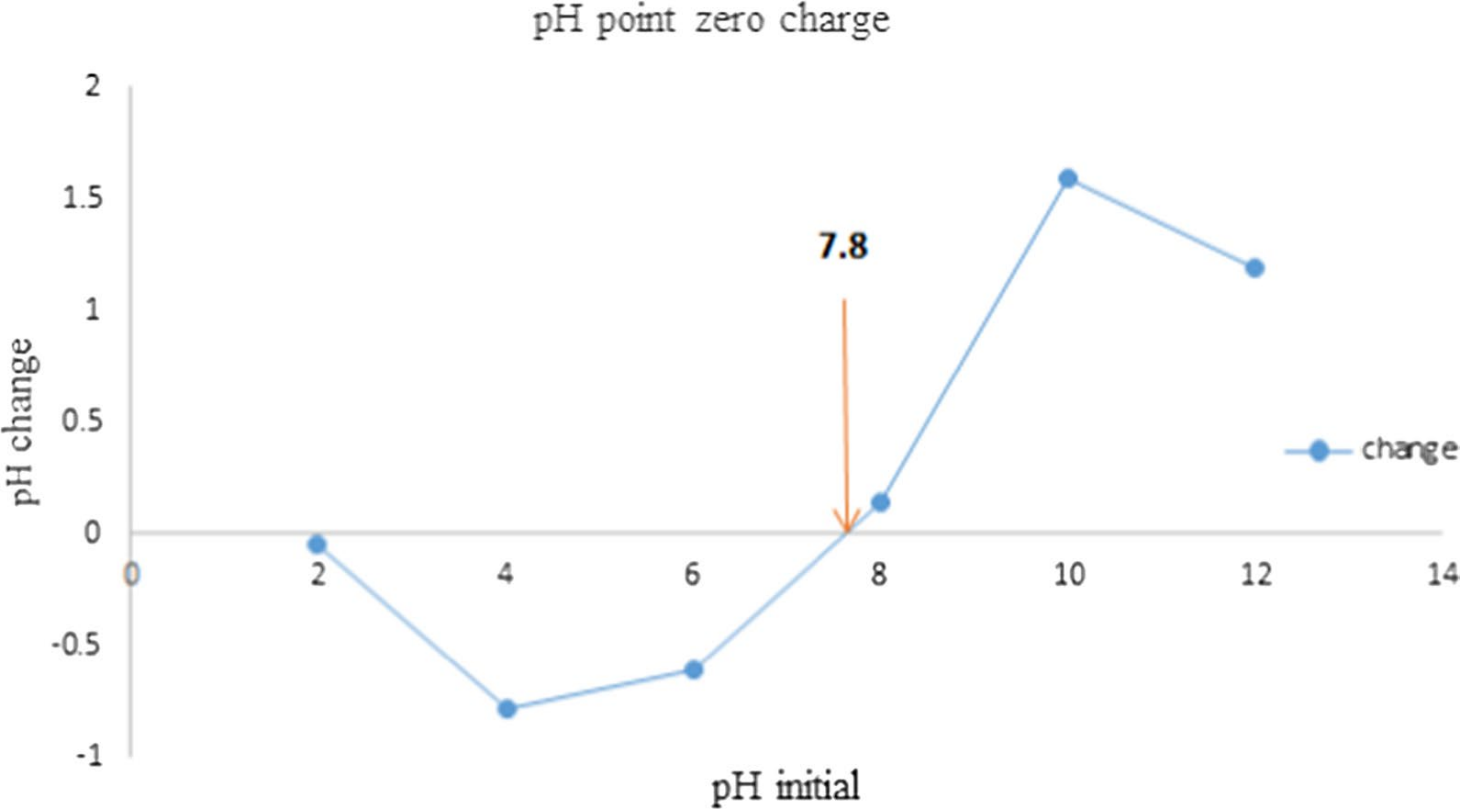
The pHpzc of activated carbon of water hyacinth

The pHpzc of activated carbon is essential to determine the surface charge of the adsorbent. This showed that the point at which the net surface charge is zero. The pHpzc of activated carbon was found to be 7.8 which is presented in Fig. 4. This point determines the degree of the interaction between the adsorbate and adsorbent material. The surface charge of the activated carbon is positive below 7.8 and completely negatively charged above this point in an aqueous solution. Hence, based on the chemical composition (functional groups) and pH of the adsorbate, the nature of interaction and the possibility of the adsorbent's effectiveness can be estimated. The adsorption effectiveness pH of anions is usually below the pHpzc whereas the cation's adsorption is above the pHpzc. The pHpzc can be found at any pH but the commercial AC powders (ACS25) 5.0, oil palm trunk-derived activated carbon 4.8, Leucaena leucocephala seed pod activated carbon 5.20, granular activated carbon 4.89, and rice husk activated carbon-supported Zink oxide 5.10 were reported [51–55].

#### BET-specific surface area

One reliable method of specific surface area evaluation was the BET method which is based on the adsorption–desorption method under nitrogen gas. After gassing the activated carbon of water hyacinth, the specific surface area was determined using BET isotherm studies. Based on this approach, the specific surface area of the adsorbent before adsorption was found to be 203.83 m^2^/g. This value of the surface area is high and has a high potential to remediate water and wastewater [28, 35]. Fundamentally, the high specific surface area, fixed carbon composition, and low ash content are the precondition for good adsorbent to interact with multifunctionality pollutants that could be found in water and wastewater systems. Even though the specific surface area is associated with the particle size which will increase with decreasing the particle size. The specific surface area of this adsorbent is superior compared to laboratory-based locally prepared activated carbon. After adsorption, the specific surface area of the adsorbent is decreased to 153.33 m^2^/g. The occupation of pores by the Cr (VI) ion might be the reason for the reduction of the specific surface area.

#### Fourier transforms infrared spectroscopy (FTIR)

**Fig. 5:**
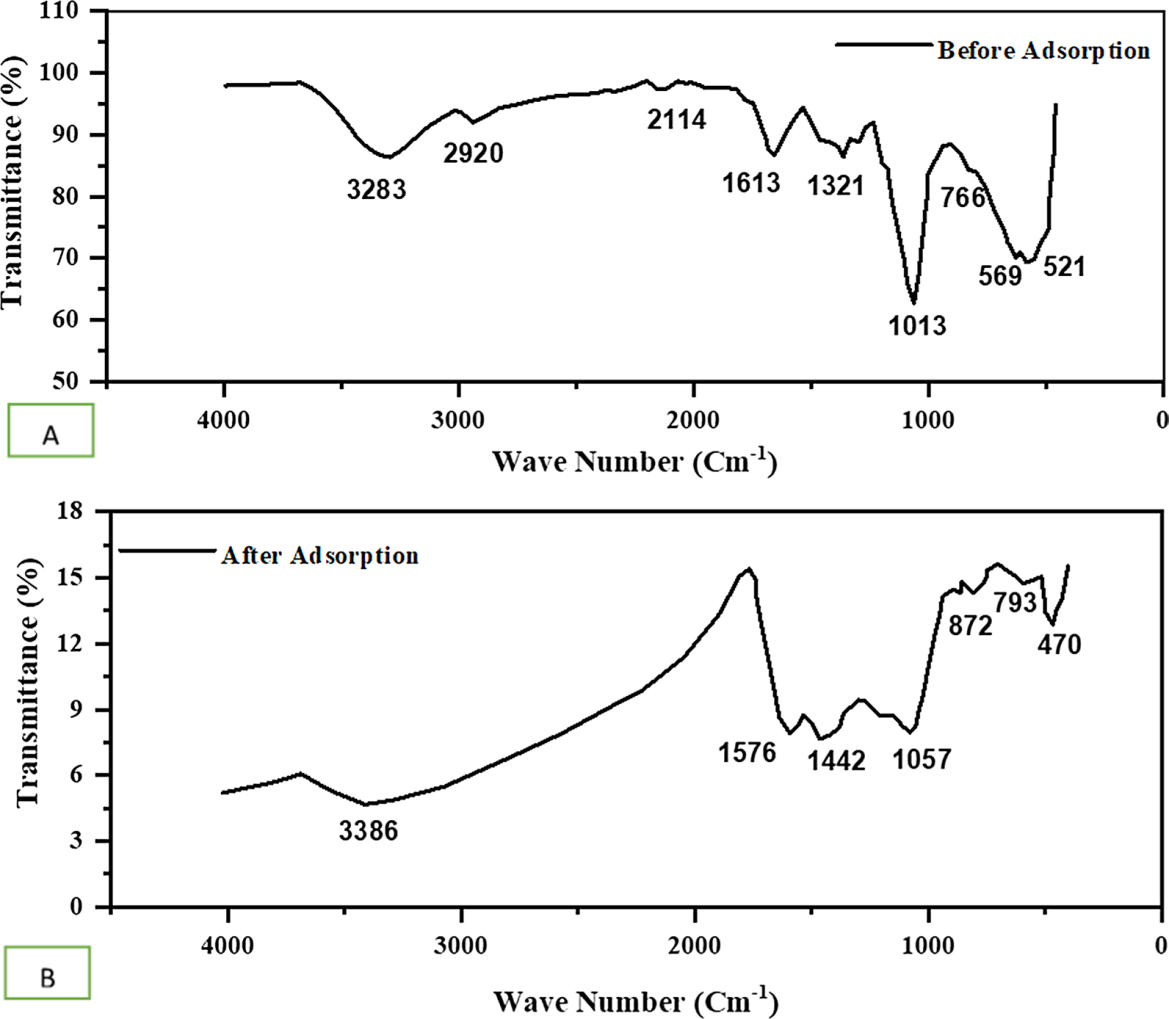
The FTIR peaks for activated carbon before adsorption **A** and after adsorption **B**

The functional groups of the activated carbon of water hyacinth were assessed using FTIR analysis. The number of the functional groups of the adsorbent is presented in Fig. 5. This adsorbent showed the presence of many functional groups which indicates the promising adsorbent to interact with many pollutants in the wastewater. The band at 3283 cm^−1^ and 3386 is attributed to O–H stretch vibration, 2920 cm^−1^ corresponds to C-H stretch vibration for alkanes, 2114 cm^–1^ attributed to non-conjugated C-N stretching vibrations, 1613 cm^−1^ and 1576 cm^−1^ correspond to C = O stretch vibration of carboxylate, 1321 cm^−1^ and 1442 cm^−1^ attributed to C-H bending vibrations (C-H deformation), 1013 cm^−1^ and 1057 cm^−1^ correspond to C-O stretches. Peaks at 766, 793, and 872 cm^−1^ are attributed to C-H bending vibration. The peaks at 569 cm^−1^ and 521 cm^−1^ correspond to phosphate groups from the phosphoric acid [28, 35, 56]. The presence of the C-O stretch indicates the alcoholic hydroxyl groups, especially the primary alcohols. These multi-functionalities corroborate the findings of the previously studied activated carbon. In addition to the high surface area, surface multi functionalities of activated carbon are good adsorbents with promising adsorption performances.

#### SEM surface morphology

**Fig. 6:**
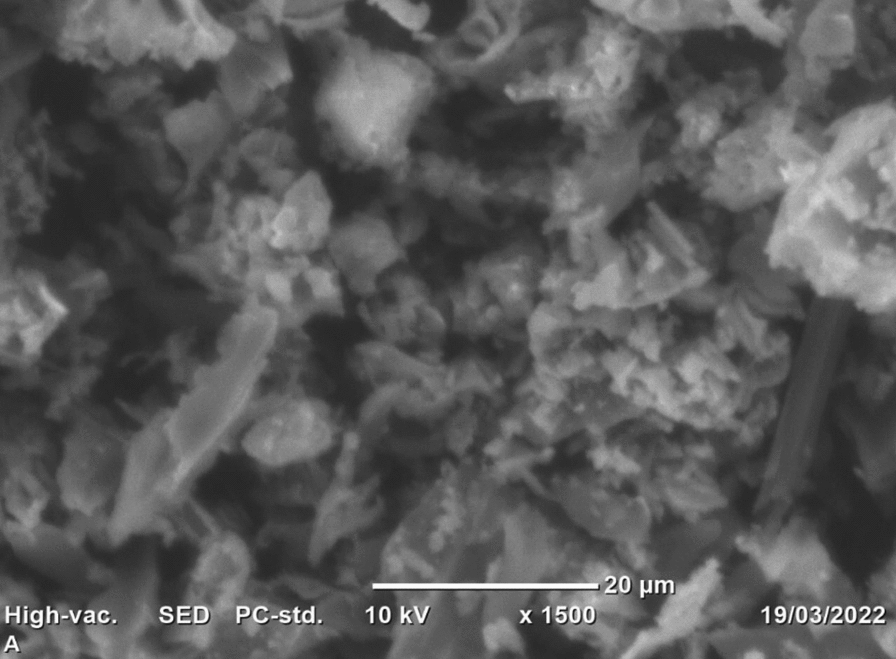
The image of SEM surface morphology of water hyacinth-activated carbon

The SEM surface morphology of the activated water hyacinth was examined and the outcome of the SEM analysis is illustrated in Fig. 6. Unevenly distributed ups and downs with huge cracks were found on the surface of the activated water hyacinth. This kind of surface morphology can create good opportunities to interact with the different varieties of pollutants. The dark surface color is attributed to the beam of electrons passage into pores whereas the bright image is possessed due to the electron beam reflected by the detector [23, 28]. Normally, large pores, roughage, and cracks on the broad spectrum of porosity with a high surface area are a good opportunity to treat high-strength and complex wastewater treatment. It was reported that larger-sized pores, non-uniform heterogeneous and irregular in shape with a lot of cracks are the indicator of plenty of binding sites for adsorbates of different sizes [46].

### Batch adsorption performance

The batch experimental results revealed that the removal efficiency of Cr ranged from 15.6 to 90.4%. These removal efficiencies were attained in the complete absence of interfering ions. The maximum removal efficiency of 90.4% (6.03 mg/g) was recorded at optimum conditions of 100 mg/L, pH 2, adsorbent dose 1.5 mg/100 mL, and contact time of 120 min as shown in Table 4. On the other hand, the minimum15.6% (3.12 mg/g) Cr (VI) removal was recorded at the experimental conditions of adsorbent dose 0.5 mg/100 mL, pH 8, contact time 30 min, and initial Cr (VI) concentration of 100 mg/L. Moreover, the maximum removal of efficiency of Cr from actual wastewater was found to be 81.4% at a Cr concentration of 92.6 mg/L. This suggests that the removal efficiencies of both real and synthetic wastewater are nearly the same. However, the maximum removal efficiency of Cr recorded at optimum conditions using raw water hyacinth was 59.2%. As per the experimental results, increasing contact time and adsorbent dosage increased the adsorption performance. However; the decreasing pattern was seen as the initial Cr concentration increased. The maximum removal efficiency recorded at pH 2 is in line with the pH point of zero charge analysis, where the adsorption of anions is expected at the positively charged surface of the adsorbent. On the other hand, reducing contact time from 120 to 30 min, and shifting the adsorbent dose from 1.5 to 0.5 keeping pH value at 2 resulted in the reduction of adsorption performance from 90.4 to 69.6 and 90.4 to 44.1, respectively. The increase in percentage adsorption with the increase in adsorbent dosage is due to the availability of more surface area of the adsorbent for adsorption. Hence, the change in contact time and adsorbent significantly affected the adsorption performance. Under the same experimental condition, shifting the pH values from 2.0 to 8.0 decreased the adsorption performance from 90.4 to 75.9, whereas varying the adsorbent dose from 0.5 to 1.5 g and contact time from 30 to 120 min resulted in increasing in adsorption performance in both cases. This clearly indicated that the shift of adsorbent dosage from the optimum point of 1.5 to 0.5 decreased the removal efficiency by 46.3%. Similarly, the shift of pH from the optimum point 2 to 8 resulted in a reduction in Cr removal efficiency of 14.5%. These results corroborate the findings of many studies [30–32] in acidic pH, the adsorbent surface may be protonated and hence the positively charged adsorbent removes the higher amount of Cr (VI) in the anionic form HCrO_4_^−^. With the increase in pH of the system, the degree of protonation of the surface reduced gradually, and hence decreased adsorption was noticed. In line with this shifting contact time from 120 to 30 min resulted in a reduction of adsorption performance of 20.8% in the previous studies [31, 32].

**Table 4:** Response surface methodology for adsorption analyses

Run	pH	Adsorbent dose(g/mL)	Contact- time(min)	Removal efficiency (%)	Adsorption capacity(g/mg)
1	5	0.5	30	21.9	4.38
2	8	0.5	30	15.6	3.12
3	8	1.5	30	44.5	2.97
4	8	1	30	33.6	3.36
5	2	1.5	30	69.6	4.64
6	2	0.5	30	22.4	4.48
7	2	0.5	75	42.5	8.5
8	2	1.5	75	87.5	5.83
9	5	0.5	75	34.9	6.98
10	5	1.5	75	77.7	5.18
11	8	1	75	49.2	4.92
12	8	1.5	75	52.5	3.5
13	5	1	75	70.4	7.04
14	2	0.5	120	44.1	8.82
15	8	1.5	120	75.9	5.06
16	8	1	120	68.5	6.85
17	5	1	120	82.5	8.25
18	5	1.5	120	88.6	5.87
19	2	1.5	120	90.4	6.03
20	8	0.5	120	43.9	8.78

### Regression and ANOVA analysis of adsorption

In this study, linear, interactive, quadratic, and cubic regression model analyses were checked. The quadratic regression model was found to fit the data well as presented in Table 5. This can be explained by Eqs. 12 and 13 quadratic equations and after insignificant terms are removed respectively. As per the statistical analysis of Cr removal from electroplating wastewater the coefficient of determination R^2^ which is 0.98 shows that 98% of the data were best fitted with the developed model. Moreover, the predicted R^2^ of 0.92 is in good agreement with the adjusted R^2^ of 0.97; i.e., the difference is 0.0452 which is less than 0.2. On the other hand, an adequacy precision value of 33.047 indicates an adequate signal. Hence this model can be used to navigate the design space. The model F value of 60.01 implies the model is significant. There is only a 0.01% chance that an F value this large could occur due to noise.

**Table 5:** ANOVA for quadratic model and its removal efficiency

Source	Sum of Squares	df	Mean Square	F-value	p-value	
Model	10,689.37	9	1187.71	60.01	< 0.0001	Significant
A-pH	814.84	1	814.84	41.17	< 0.0001	
B-adsorbent dos	5146.30	1	5146.30	260.03	< 0.0001	
C-contact time	2199.30	1	2199.30	111.13	< 0.0001	
AB	213.44	1	213.44	10.78	0.0082	
AC	59.90	1	59.90	3.03	0.1125	
BC	0.0177	1	0.0177	0.0009	0.9767	
A^2^	128.09	1	128.09	6.47	0.0292	
B^2^	288.18	1	288.18	14.56	0.0034	
C^2^	6.19	1	6.19	0.3126	0.5884	
Residual	197.91	10	19.79			
Cr Total	10,887.28	19				

P-values less than 0.0500 and greater than 0.1 indicate model terms that are significant and insignificant respectively. In this case, A, B, C, AB, A^2^, and B^2^ are significant model terms whereas C^2^, AC, and BC are found to be insignificant. Normally many insignificant terms are not required in regression analysis and lead to a model reduction. The developed model predicts the removal efficiency of 93.7% at optimum conditions of pH 2, contact time 120 min, and adsorbent dosage of 1.5 g/100 mL. This predicted value is nearly equal to the experimentally determined value of 90.4% with a standard deviation of 3.5.

12 $$67.48 - 8.40{\text{A + 19}}{\text{.08B + 13}}{\text{.61C}} - {4}{\text{.50AB + 2}}{\text{.49AC}} - 0.0444{\text{BC}} - 5.97{\text{A}}^{2} - 9.678{\text{B}}^{2} - 1.23{\text{C}}^{2}$$

13 $$67.48 - 8.40{\text{A + 19}}{\text{.08B + 13}}{\text{.61C}} - 4.50{\text{AB}} - 5.97{\text{A}}^{2} - 9.67{\text{B}}^{2}$$

**Fig. 7:**
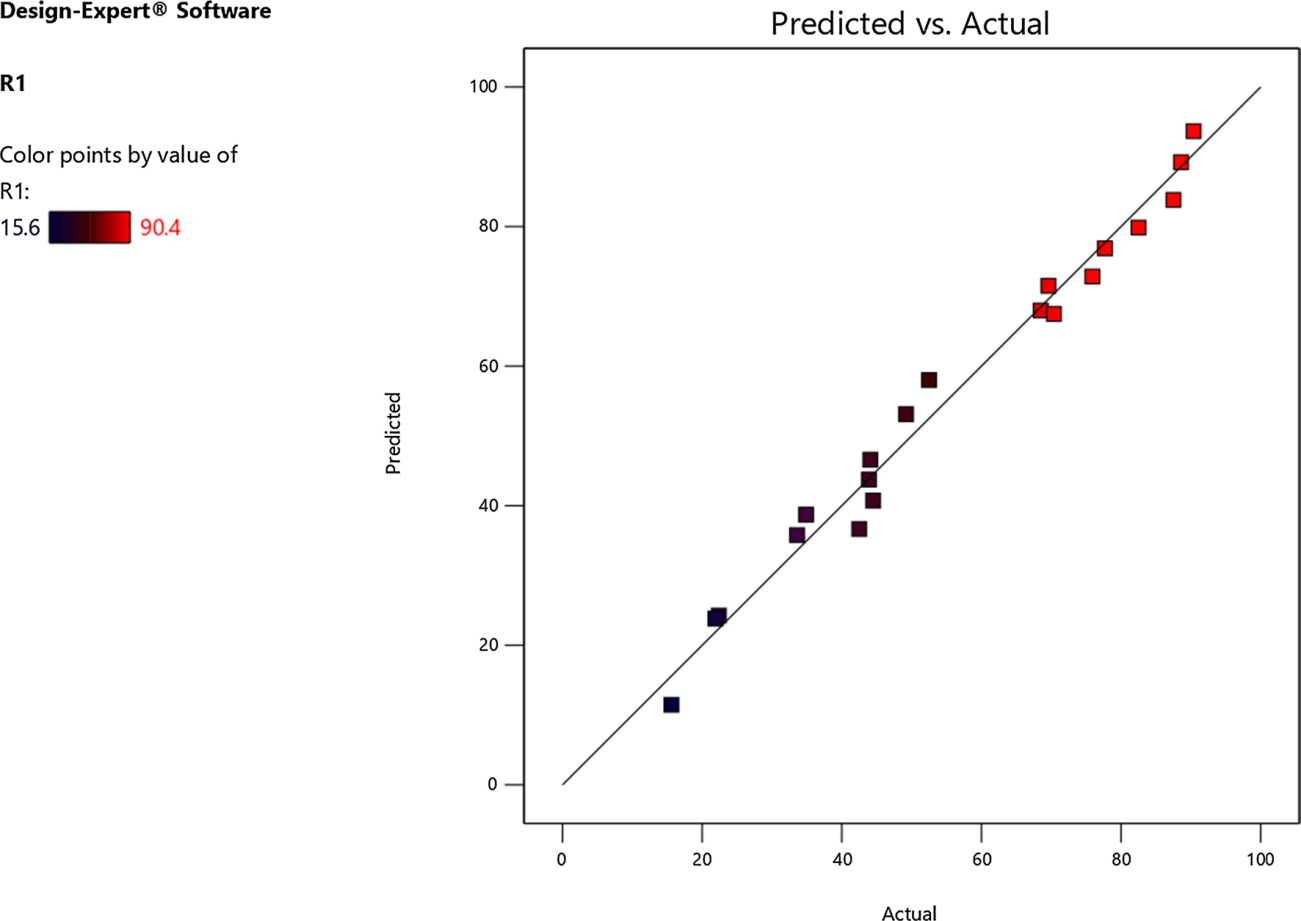
Predicted and actual values for Cr removal efficiency

The graphical representation of predicted and actual values is presented in Fig. 7. It can be observed from the graph that both predicted and actual values are distributed around the linear line passing through the origin showing the error distribution normal.

### Interaction effects

#### Adsorbent dosage and pH

**Fig. 8:**
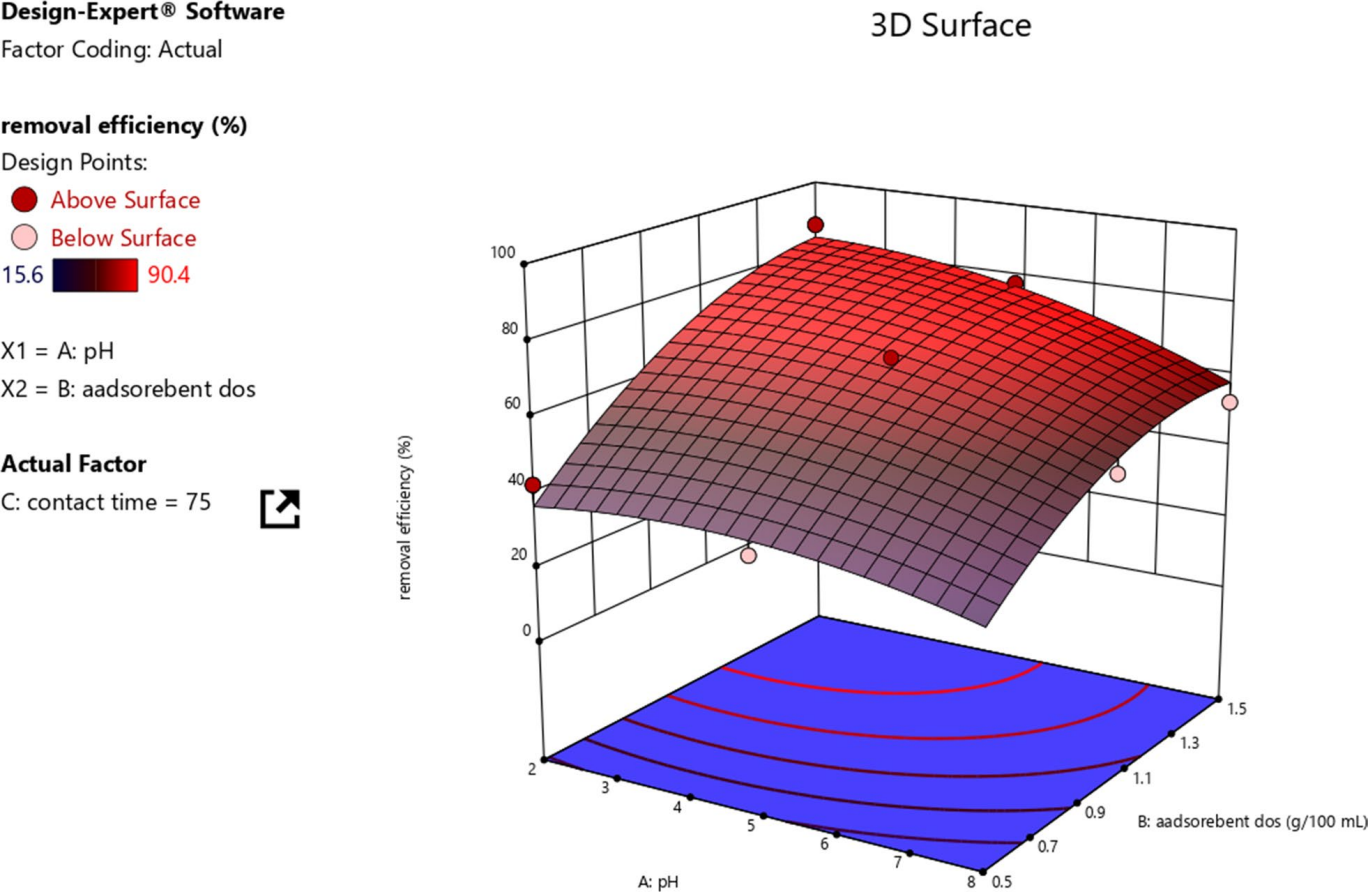
The interaction effect of Adsorbent dosage and pH on Cr removal

The 3D plot of adsorbent dosage, pH, and Cr removal efficiency was used to evaluate the interactions of pH and adsorbent dosage on the Cr removal efficiency. Figure 8 shows that the removal efficiency ranged from 15.6% to 90.4% indicated on the 3D plot. Normally, the interaction effect was determined by keeping the contact time constant at the middle value (75 min) while the pH and adsorbent dosage varied from 2 to 8 and 0.5 to 1.5 g/100 mL, respectively. It can be observed from the graph that the interaction was found to be negative. Hence, the removal efficiency of Cr from electroplating wastewater was negatively affected by the interaction of pH and dosage. Individually, increasing pH decreases the removal efficiency whereas increasing adsorbent dosage favored the adsorption process. Hence, the effect of pH was high compared to the adsorbent dosage.

#### Solution pH and contact time

**Fig. 9:**
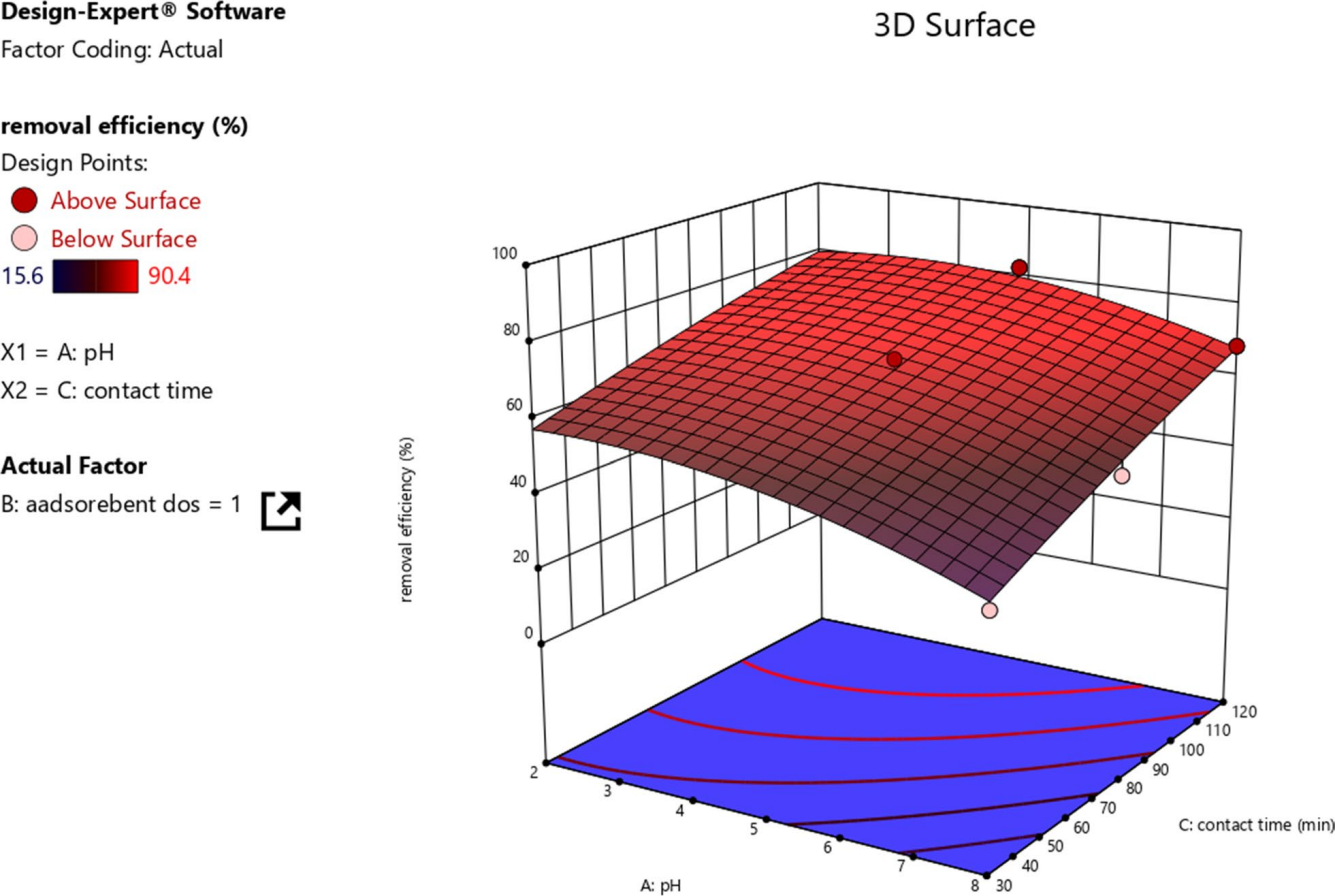
The interaction effect of solution pH and contact time on Cr removal

The interaction effect of pH and contact time was studied using response surface methodology. The 3D graph was plotted using pH, and contact time with Cr removal efficiency is shown in Fig. 9. The interaction effect was determined by keeping the adsorbent dosage constant at 1 g/100 mL. The interaction was determined to have a favorable impact on the removal efficiency of Cr. However, individually increasing pH and contact time decrease and favors the removal efficiency, respectively. Compared to the solution pH, the effect of contact time was found to be significant. Hence, contact time highly determines both the removal efficiency and adsorption capacities.

#### Adsorbent dosage and contact time

**Fig. 10:**
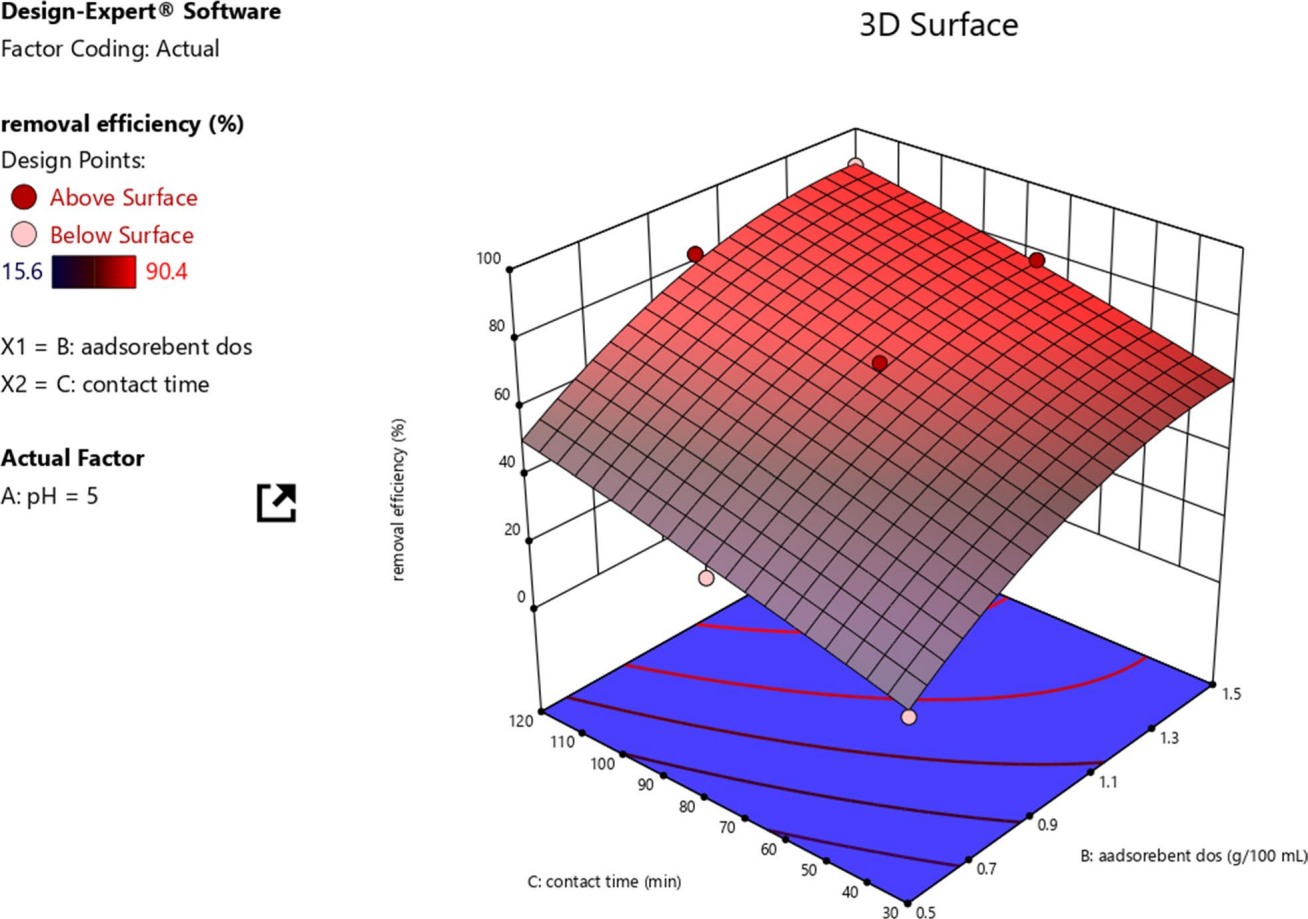
The interaction effect of adsorbent dosage and contact time on Cr removal

The interaction effect of the adsorbent dose and contact time on Cr (VI) removal was investigated and the interaction effect on the Cr removal performance is shown in Fig. 10. The response surface plot is used to describe the influence of the interaction on the response variable through the 3D graph. This graph indicates the variable pattern of Cr (VI) removal using activated carbon of water hyacinth. The predicted treatment performances were nearly the same as the actual ones. This indicates that the predicted model had the goodness of fit with the experimental value. The removal efficiency of Cr (VI) was negatively influenced by the interaction effect of adsorbent dosage and contact time, showing the maximum Cr (VI) removal of 90.4% was achieved at the interaction of 1.5 g adsorbent dosage and 120 min contact time.

### Adsorption isotherms

The experimental data were fitted with both the Langmuir and Freundlich models. Based on the findings, the best data fit is inclined to the Langmuir isotherm model indicating the interaction of adsorbent and adsorbent monolayer and homogenous surface as shown in Table 6. As per Langmuir's isotherm, the coefficient determinant R^2^ was found to be 0.96. Moreover, the Langmuir maximum adsorption capacity was determined to be 2.63*10^–4^ mg/g. Similarly, the RL value of 0.003 lying between 0 and 1 indicates that the adsorption process is favorable. However, the Freundlich isotherm plot at R^2^ 0.86 was not illustrated in Table 6. The *n* value of Freundlich isotherm was 3.18, reconfirming the favourability of adsorption of Cr onto activated carbon of water hyacinth.

**Table 6:** Adsorption isotherm models

Isotherm model		
Language	R^2^	0.9597
	qm (mg/g)	2.63*10^–4^
	Kl (L/mg)	166.34
	Equation	3800.2 X + 22.859
Fredulich	R^2^	0.8564
	n	− 0.575
	Kf ((mg/g)(L/mg)1/n	5063.93
	Equation	− 1.7395 X + 8.5299

### Adsorption kinetics

Adsorption kinetics studies evaluate the time required for the movement of the system from an initial to a final state. As shown in Table 7, the pseudo-second-order model with an R^2^ value of 0.99 best fits with the experimental data compared with the pseudo-first-order model with an R^2^ value of 0.56. This indicates that chemical reaction controls the adsorption kinetics.

**Table 7:** Adsorption Kinetics

Kinetics model		
Pseudo-first-order	R^2^	0.5556
	K_l_ (min^−1^)	0.014
	Equation	Y = 0.006 X + 3.323
Pseudo-second-order	R^2^	0.9891
	K_2_ (g/mg/min)	3.21*10^–5^
	Equation	Y = 0.0003X + 0.0028

### Regeneration study

**Fig. 11:**
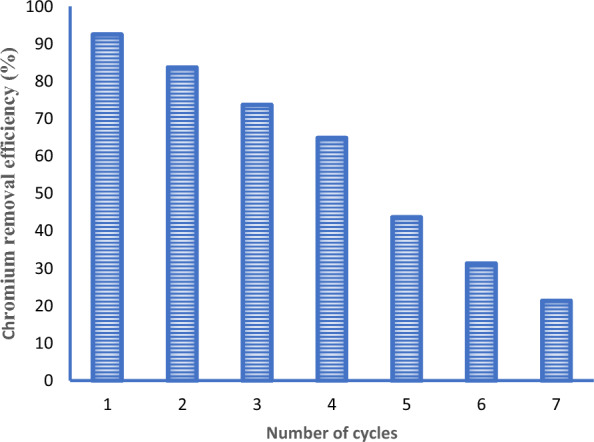
Regeneration study

The removal efficiency of the adsorbent decreased from 93.25% to 21.35% as we go from the first cycle to the seventh cycle as shown in Fig. 11. The reduction of the removal efficiencies could be due to the loss of materials and the changes in the adsorbent properties during the regeneration process such as aggregation and fouling.

The comparison of different removal efficiency is indicated in Table 8. However, the results of the findings are closely the same but the condition of the experiment and the concentration of the pollutants are quite different.

**Table 8:** Comparison of this study with others

Precursor material	Removal efficiency	References
*Eichhornia crassipes*	89.9%	[57]
Cellulose of Eichhornia crassipes	122.2 mg/g	[58]
Eichhornia crassipes root	36.3 mg/g	[59]
Water hyacinth roots	95.4%	[60]
Eichhornia crassipes	84–99%	[61]
Eichhornia crassipes	96.4%	[62]
Eichhornia crassipes Biochar	27.3–3190.1 mg/L	[63]
Eichhornia crassipes	98.4%	[64]
*Eichhornia crassipes*	90.40	This study

## Conclusions

High concentrations of the physicochemical parameters of electroplating wastewater were found which required urgent treatment intervention. Specifically, a high Cr concentration (92.16 ± 0.768 mg/L) was obtained in electroplating industrial wastewater. Mainly, this study aimed to remove Cr ions from electroplating wastewater using activated carbon produced from water hyacinth under a full factorial experimental design with three factors and three levels (pH,2,5and8, adsorbent dose 0.5,1and1.5 in 100 mL and contact time 30, 60 and120 min). This adsorbent was activated chemically and thermally and its characteristics were described in terms high of BET surface area (203.83 m^2^/g, ups and down (cracks) of morphology, and multi functionalities of hydroxyl (3283 cm^−1^), alkane (2920 cm^−1^), nitrile (2114 cm^−1^) and aromatics (1613 cm^−1^). The properties of this adsorbent are promising to scale up the application at the industrial level. After adsorption, the BET surface area decreased to 153.33 m^2^/g. The maximum removal of 90.4% was recorded at the experimental condition of pH 2, adsorbent dose of 1.5 g/100 mL, and contact time of 120 min at a fixed value of initial chromium concentration of 100 mg/L whereas the maximum Cr removal from real electroplating wastewater was found to be 81.2%. Among the two adsorption isotherms applied to investigate the adsorption mechanism, the Langmuir isotherm best described the experimental values at R^2^ 0.96 indicating that the adsorption process was homogeneous and monolayer. Pseudo-second-order kinetics model with an R^2^ value of 0.99 best fits the experimental data indicating that chemical reaction controls the adsorption kinetics. The adsorbent was regenerated for seven cycles and the removal efficiency decreased from 93.25% to 21.35%. This activated carbon is a promising technology for the removal of Cr removal from electroplating wastewater. However, further adsorbent investigations in terms of adsorption kinetics, optimization process, and thermodynamics are highly recommended.

## Data Availability

All data are fully available without restriction from the corresponding author.

## References

[1] UN-water (2021) Valuing water,The United nations world water development report 2021,the United Nations educational scientific and cultural organization 7 place de Fontenoy, 75352 Paris 07 SP, France.

[2] Timmers PHA, Slootweg T, Knezev A (2022). Improved drinking water quality after adding advanced oxidation for organic micropollutant removal to pretreatment of river water undergoing dune infiltration near the Hague Netherlands. J Hazard Mater.

[3] Aemig Q, Hélias A, Patureau D (2021). Impact assessment of a large panel of organic and inorganic micropollutants released by wastewater treatment plants at the scale of France. Water Res.

[4] Mahy JG, Wolfs C, Mertes A (2019). Advanced photocatalytic oxidation processes for micropollutant elimination from municipal and industrial water. J Environ Manage.

[5] Peña-Guzmán C, Ulloa-Sánchez S, Mora K (2019). Emerging pollutants in the urban water cycle in Latin America: a review of the current literature. J Environ Manage.

[6] Bhatt P, Bhandari G, Bilal M (2022). Occurrence, toxicity impacts and mitigation of emerging micropollutants in the aquatic environments: Recent tendencies and perspectives. J Environ Chem Eng.

[7] Bankole MT, Abdulkareem AS, Mohammed IA (2019). Selected heavy metals removal from electroplating wastewater by purified and polyhydroxylbutyrate functionalized carbon nanotubes adsorbents. Sci Rep.

[8] Shan H, Zeng C, Zhao C, Zhan H (2021). Iron oxides decorated graphene oxide/chitosan composite beads for enhanced Cr(VI) removal from aqueous solution. Int J Biol Macromol.

[9] Sathish M, Dhathathreyan A, Rao JR (2019). Ultra efficient tanning process: role of mass transfer efficiency and sorption kinetics of Cr(III) in leather processing. ACS Sustain Chem Eng.

[10] Liu X, Wang Y, Wang X (2022). A salt-free pickling and chrome-free tanning technology: a sustainable approach for cleaner leather manufacturing. Green Chem.

[11] Zhou R, Jin Y, Lai S (2022). A novel composite retanning system based on pH-responsive hydrogen bonding and hydrophobic interaction for cleaner leather processing. J Clean Prod.

[12] Yadav A, Raj A, Purchase D (2019). Phytotoxicity, cytotoxicity and genotoxicity evaluation of organic and inorganic pollutants rich tannery wastewater from a common effluent treatment plant (CETP) in Unnao district, India using Vigna radiata and Allium cepa. Chemosphere.

[13] Huang Y, Lee X, Macazo FC (2018). Fast and e ffi cient removal of chromium ( VI ) anionic species by a reusable chitosan-modified multi-walled carbon nanotube composite. Chem Eng J.

[14] Aigbe UO, Osibote OA (2020). A review of hexavalent chromium removal from aqueous solutions by sorption technique using nanomaterials. J Environ Chem Eng.

[15] Kong D, He L, Li H (2021). Preparation and characterization of graphene oxide/chitosan composite aerogel with high adsorption performance for Cr(VI) by a new crosslinking route. Colloids Surfaces A Physicochem Eng Asp.

[16] Preethi J, Prabhu SM, Meenakshi S (2017). Effective adsorption of hexavalent chromium using biopolymer assisted oxyhydroxide materials from aqueous solution. React Funct Polym.

[17] Zhao C, Chen W (2019). A review for tannery wastewater treatment: some thoughts under stricter discharge requirements. Environ Sci Pollut Res.

[18] Evers M, Lange R-L, Heinz E, Wichern M (2022). Simultaneous powdered activated carbon dosage for micropollutant removal on a municipal wastewater treatment plant compared to the efficiency of a post treatment stage. J Water Process Eng.

[19] Yacouba ZA, Mendret J, Lesage G (2021). Removal of organic micropollutants from domestic wastewater: The effect of ozone-based advanced oxidation process on nanofiltration. J Water Process Eng.

[20] Fito J, Tefera N, Kloos H, Van Hulle SWH (2018). Anaerobic treatment of blended sugar industry and ethanol distillery wastewater through biphasic high rate reactor. J Environ Sci Heal Part A Toxic/Hazardous Subst Environ Eng.

[21] Jahangiri FM, Moutushi HT, Moniruzzaman M (2021). Removal of lead from aqueous solutions and wastewaters using water hyacinth (*Eichhornia* crassipes) roots. Water Pract Technol.

[22] Badessa TS, Wakuma E, Yimer AM (2020). Bio-sorption for effective removal of chromium (VI) from wastewater using Moringa stenopetala seed powder (MSSP) and banana peel powder (BPP). BMC Chem.

[23] Moges A, Nkambule TTI, Fito J (2022). The application of GO-Fe 3 O 4 nanocomposite for chromium adsorption from tannery industry wastewater. J Environ Manage.

[24] Madhuranthakam CMR, Thomas A, Akhter Z (2021). Removal of chromium (VI ) from contaminated water using untreated moringa leaves as biosorbent. Pollutants.

[25] Varsha M, Senthil Kumar P, Senthil Rathi B (2022). A review on recent trends in the removal of emerging contaminants from aquatic environment using low-cost adsorbents. Chemosphere.

[26] Singh NB, Nagpal G, Agrawal S (2018). Water purification by using adsorbents: a review. Environ Technol Innov.

[27] Fito J, Kefeni KK, Nkambule TTI (2022). The potential of biochar-photocatalytic nanocomposites for removal of organic micropollutants from wastewater. Sci Total Environ.

[28] Bedada D, Angassa K, Tiruneh A (2020). Chromium removal from tannery wastewater through activated carbon produced from *Parthenium* hysterophorus weed. Energy Ecol Environ.

[29] Rani N, Singh B, Shimrah T (2017). Chromium (VI) removal from aqueous solutions using Eichhornia as an adsorbent. J Water Reuse Desalin.

[30] Gude SM, Das SN (2008). Adsorption of chromium (VI) from aqueous solutions by chemically treated water hyacinth Eichhornia crassipes. Ind J Chem Technol.

[31] Yang J, Yu M, Chen W (2015). Adsorption of hexavalent chromium from aqueous solution by activated carbon prepared from longan seed: Kinetics, equilibrium and thermodynamics. J Ind Eng Chem.

[32] Santhosh P, Dhandapani C (2013). Adsorption studies on the removal of chromium (vi) from wastewater using activated carbon derived from water hyacinth. Nature Environ Poll Technol.

[33] Neris JB, Luzardo FHM, Santos PF (2019). Evaluation of single and tri-element adsorption of Pb 2+, Ni 2+ and Zn 2+ ions in aqueous solution on modified water hyacinth (*Eichhornia* crassipes) fibers. J Environ Chem Eng.

[34] Kabir MM, Alam F, Akter MM (2022). Highly effective water hyacinth (Eichhornia crassipes) waste-based functionalized sustainable green adsorbents for antibiotic remediation from wastewater. Chemosphere.

[35] Bapat SA, Jaspal DK (2020). Surface-modified water hyacinth (*Eichhornia* crassipes) over activated carbon for wastewater treatment: a comparative account. South African J Chem.

[36] Liu C, Ye J, Lin Y (2020). Removal of cadmium (II) using water hyacinth (*Eichhornia* crassipes) biochar alginate beads in aqueous solutions. Environ Pollut.

[37] Carreño-Sayago UF (2021). Development of microspheres using water hyacinth (*Eichhornia* crassipes) for treatment of contaminated water with Cr(VI). Environ Dev Sustain.

[38] Ramirez-Muñoz A, Pérez S, Flórez E, Acelas N (2021). Recovering phosphorus from aqueous solutions using water hyacinth (*Eichhornia* crassipes) toward sustainability through its transformation to apatite. J Environ Chem Eng.

[39] Hashem MA, Hasan M, Momen MA (2020). Water hyacinth biochar for trivalent chromium adsorption from tannery wastewater. Environ Sustain Indic.

[40] Waktole Y, Seid B, Mereta T, Fufa F (2019). Simultaneous removal of nitrate and phosphate from wastewater using solid waste from factory. Appl Water Sci.

[41] Yehuala G, Worku Z, Angassa K (2021). Electrochemical degradation of chemical oxygen demand in the textile industrial wastewater through the modified electrodes. Arab J Sci Eng.

[42] Fito J, Abrham S, Angassa K (2020). Adsorption of methylene blue from textile industrial wastewater onto activated carbon of parthenium hysterophorus. Int J Environ Res.

[43] Hameed BH, Tan IAW, Ahmad AL (2008). Adsorption isotherm, kinetic modeling and mechanism of 2,4,6-trichlorophenol on coconut husk-based activated carbon. Chem Eng J.

[44] Hegazy AK, Abdel-Ghani NT, El-Chaghaby GA (2014). Adsorption of phenol onto activated carbon from *Rhazya* stricta: determination of the optimal experimental parameters using factorial design. Appl Water Sci.

[45] Tebeje A, Worku Z, Nkambule TTI, Fito J (2021). Adsorption of chemical oxygen demand from textile industrial wastewater through locally prepared bentonite adsorbent. Int J Environ Sci Technol.

[46] Nure JF, Shibeshi NT, Asfaw SL (2017). COD and colour removal from molasses spent wash using activated carbon produced from bagasse fly ash of matahara sugar factory, oromiya region, Ethiopia. Water SA.

[47] Caicedo C, Rosenwinkel KH, Exner M (2019). Legionella occurrence in municipal and industrial wastewater treatment plants and risks of reclaimed wastewater reuse: review. Water Res.

[48] Balomajumde BVC (2020). Hexavalent chromium reduction from real electroplating. Bull Chem Soc Ethiopia.

[49] Maulina S, Iriansyah M. Characteristics of activated carbon resulted from pyrolysis of the oil palm fronds powder. In: IOP conference series: materials science and engineering. IOP Publishing, p 12072. 2018.

[50] Fito J, Said H, Feleke S, Worku A (2019). Fluoride removal from aqueous solution onto activated carbon of Catha edulis through the adsorption treatment technology. Environ Syst Res.

[51] Hsu CC, Tu YH, Yang YH (2020). Improved performance and long-term stability of activated carbon doped with nitrogen for capacitive deionization. Desalination.

[52] Lim A, Chew JJ, Ngu LH (2020). Synthesis, characterization, adsorption isotherm, and kinetic study of oil palm trunk-derived activated carbon for tannin removal from aqueous solution. ACS Omega.

[53] Yusuff AS (2019). Adsorption of hexavalent chromium from aqueous solution by *Leucaena* leucocephala seed pod activated carbon: equilibrium, kinetic and thermodynamic studies. Arab J basic Appl Sci.

[54] Rashid US, Bezbaruah AN (2020). Citric acid modified granular activated carbon for enhanced defluoridation. Chemosphere.

[55] Pramanik SK, Suja FB, Zain S, Pramanik BK (2019). Journal pre. Bioresour Technol Reports.

[56] Ibrahim M, Osman O, Mahmoud AA, Elhaes H (2015). Spectroscopic analyses of water hyacinth: FTIR and modeling approaches. Der Pharma Chem.

[57] Wei Y, Fang Z, Zheng L, Tsang EP (2017). Biosynthesized iron nanoparticles in aqueous extracts of *Eichhornia* crassipes and its mechanism in the hexavalent chromium removal. Appl Surf Sci.

[58] Tan L, Zhu D, Zhou W (2008). Preferring cellulose of *Eichhornia* crassipes to prepare xanthogenate to other plant materials and its adsorption properties on copper. Bioresour Technol.

[59] Giri AK, Patel R, Mandal S (2012). Removal of Cr (VI) from aqueous solution by *Eichhornia* crassipes root biomass-derived activated carbon. Chem Eng J.

[60] Kumar P, Chauhan MS (2019). Adsorption of chromium (VI) from the synthetic aqueous solution using chemically modified dried water hyacinth roots. J Environ Chem Eng.

[61] Gupta A, Balomajumder C (2015). Removal of Cr(VI) and phenol using water hyacinth from single and binary solution in the artificial photosynthesis chamber. J Water Process Eng.

[62] Hemalatha D, Narayanan RM, Sanchitha S (2020). Removal of Zinc and chromium from industrial wastewater using water hyacinth (E. crassipes) petiole, leaves and root powder: equilibrium study. Mater Today Proc.

[63] Chen XL, Li F, Xie XJ (2019). Nanoscale zero-valent iron and chitosan functionalized *Eichhornia* crassipes biochar for efficient hexavalent chromium removal. Int J Environ Res Public Health.

[64] Fito J, Tibebu S, Nkambule TTI (2023). Optimization of Cr (VI) removal from aqueous solution with activated carbon derived from *Eichhornia* crassipes under response surface methodology. BMC Chem.

